# Blood lipid levels, statin therapy and the risk of intracerebral hemorrhage

**DOI:** 10.1186/s12944-016-0213-8

**Published:** 2016-03-01

**Authors:** Yingxu Ma, Zhaokai Li, Liang Chen, Xiangping Li

**Affiliations:** Department of Cardiology, The Second Xiangya Hospital, Central South University, #139 Middle Renmin Road, Changsha, Hunan 410011 PR China; The Eight-Year Clinical Medicine of Grade 2012, Xiangya School of Medicine, Central South University, Changsha, Hunan 410013 PR China

**Keywords:** Lipids, Statin, Lipid-lowering therapy, Intracerebral hemorrhage

## Abstract

Dyslipidemia has been proven to play an important role in the occurrence and development of the ischemic stroke and lipid-lowering therapy could significantly decrease the risk of the ischemic stroke. However, the association between lipid levels, lipid-lowering therapy and the risk of intracerebral hemorrhage (ICH) is not clear. Studies have shown that low serum levels of total cholesterol might be associated with increasing risk of ICH, whereas the SPARCL study, a large prospective, randomized, placebo-controlled trial, demonstrated an increased risk of hemorrhagic stroke during high-dose statin therapy among the patients with previous stroke. The relationship between lipid-lowering therapy and ICH has become a hot topic in the recent years. We searched PubMed for articles published in English to review the existing evidence on the association of lipid levels, statin therapy and risk of ICH as well as the underlying mechanisms in order to provide practical recommendations for clinical decision-making and a foundation for further researches.

## Background

Stroke is one of the leading causes of death and adult disability in the world. Intracerebral hemorrhage (ICH), an important subtype of the stroke, is characterized by high mortality and morbidity, which contains symptomatic intracerebral hemorrhage (sICH) [1] and cerebral microbleed (CMB) [2]. In ICH patients, perihematomal inflammation where the region becomes infiltrated with neutrophils and activated microglia after the activation of Toll-like receptor 4 [3] and the release of inflammatory mediators such as tumor necrosis factor-α (TNF-α) and interleukin-1β (IL-1β) [4] contributes to neuronal injury and functional disability. Meanwhile, ICH results in the change of the cerebral blood flow and the increasing permeability of blood–brain barrier (BBB) [5]. These pathological changes could aggravate nerve damage and dysfunction. The data from World Health Organization (WHO) showed that ICH accounts for approximately 25–50 % of stroke and the reduction of morbidity and mortality among ICH patients must remain a public health priority.

Hyperlipidemia has been proven to be a risk factor of ischemic stroke [6]. Statins which mainly reduce the serum levels of total cholesterol (TC) and low density lipoprotein cholesterol (LDL-C) are widely used or prescribed for the primary and secondary prevention of ischemic strokes and have achieved favourable clinical outcomes. Several clinical studies [1, 7–9] have shown that statin could not increase the risk of ICH and has been beneficial to ICH patients as well as promoting their recovery. But a post hoc analysis of the SPARCL trial that was conducted among the patients with stroke demonstrated that atorvastatin was associated with an increased risk of ICH [10]. Furthermore, some epidemiological and case–control studies found that patients with lower serum lipid had an increasing risk of ICH [11–13]. Therefore, it is necessary to further clarify the association of serum lipid levels, statin therapy and the risk of ICH. The systematic review focuses on the research progress in this field derived from epidemiological and clinical evidence. We searched PubMed for basic and clinical studies which were published in English (last search update performed on 31 August 2015). The search strategy was based on the combination of the following terms: lipids, total cholesterol (TC), triglyceride (TG), low density lipoprotein cholesterol (LDL-C), and high density lipoprotein cholesterol (HDL-C), lipid-lowering therapy, statins, HMG-CoA reductase inhibitors, stroke and intracerebral hemorrhage.

## Blood lipid levels and ICH

Lipids are essential components of cell membrane, which play an important role in maintaining the stabilization of endothelial cells and the integrity of cerebral small vessels [14]. Epidemiological and case–control studies found a correlation between ICH and the change of blood lipid levels which included TC, TG, LDL-C, and HDL-C.

### TG and ICH

A pooled cohort study of the Atherosclerosis Risk in Communities Study (ARIC) and the Cardiovascular Health Study (CHS) involving 21,680 patients showed that lower TG was a risk factor for ICH and was inversely related to the incidence of ICH [15]. In a population-based prospective cohort study among 8393 men and women [11], Bonaventure et al. made a quantitative analysis of TG level and the risk of ICH, demonstrating that a low level of triglycerides (serum TG ≤0.94 mmol/L) was associated with a more than two-fold increased risk of ICH [adjusted hazard ratio (HR) 2.35, 95 % confidence interval (CI) 1.18–4.70]. In addition, the Rotterdam Study showed that serum TG levels were associated with the presence of CMB [16], which is a risk marker of sICH [2]. The above results suggest that the low TG might be related to the increasing risk of ICH. On the contrary, in the Malmö Preventive Project [17] involved 33,346 participants (mean age 47 years old), 147 ICH subjects who were followed up (approx. 14 years) had significantly higher TG levels (1.7 vs. 1.4 mmol/L) compared with 1029 stroke-free controls, matched for age, sex and screening-year. The multivariate analyses showed there was a positive correlation between TG and the risk of ICH among the lobar ICH (OR 1.7, 95 % CI 0.9–3.2) and nonlobar ICH (OR 1.4, 95 % CI 0.90–2.3) patients. Furthermore, a cross-sectional study [18] demonstrated that 500 patients with acute ICH had significantly higher TG levels (*P* < 0.0001). The linear regression and correlation analysis suggested that with increased TG, the intracerebral hemorrhagic volumes were gradually increasing.

### TC and ICH

In a case–control study in 22 countries (the INTERSTROKE study) [13], with blood analyses completed among 2190 cases and 2127 controls who matched for age and sex, researchers found that increased concentration of TC was associated with reduced risk of ICH (OR 0.62, 99 % CI 0.42–0.92). A meta-analysis [19] that included 23 prospective studies, totaling 1,430,141 participants with 7960 hemorrhagic strokes (0.56 %) demonstrated that the summary relative risk of hemorrhagic stroke was 0.69 (95 % CI 0.59–0.81) in high versus low TC analysis. Meanwhile, the summary relative risk of hemorrhagic stroke for 1 mmol/L increment of TC was 0.85 (95 % CI 0.80–0.91), supporting that TC was inversely related to ICH. And Suzuki et al. [20] found that serum TC <160 mg/dl correlated with the significantly increased risk of ICH. Furthermore, the Framingham Heart Study [2] comprised 1965 Framingham residents (mean age 66.5 ± 11.0 years old; 54 % women) who attended a baseline brain MRI between 1998 and 2008 and underwent it again between 2000 and 2009. The result revealed that 8.8 % of participants had a CMB between 2000 and 2009. Logistic regression analysis [2] showed that TC < 10th percentile remained associated with the increasing risk of CMB (OR 1.9; 95 % CI 1.20–3.03). However, a prospective study [12] including 58,235 Finnish participants (age range 25–74 years old) without a history of coronary heart disease and stroke demonstrated that, after further adjustment for other confounding factors, the inverse association between TC and ICH is only significant in women but not in men.

### LDL-C and ICH

The meta-analysis conducted by Wang et al. [19] showed that the summary relative risk of hemorrhagic stroke for 1 mmol/L increment of LDL-C was 0.90 (95 % CI 0.77–1.05), suggesting that high level of LDL-C may reduce the risk of hemorrhagic stroke. Moreover, Mustanoja et al. [21] found that after adjusting for known ICH prognostic factors lower LDL-C levels was independently associated with in-hospital mortality of ICH patients (OR 0.54, 95 % CI 0.31–0.93).

### HDL-C and ICH

A case–control study [13] showed that the level of HDL-C positively correlated with the risk of ICH (OR 1.91, 99 % CI 1.29–2.83). In addition, higher ratio of non-HDL-C to HDL-C was associated with reduced risk of ICH (OR 0.43, 99 % CI 0.30–0.62) [13]. A meta-analysis [19] of 19 prospective cohort studies also indicated that the summary relative risk of hemorrhagic stroke for 1 mmol/L increment of HDL-C was 1.17 (95 % CI 1.02–1.35) in a dose–response analysis, which suggested that the increased level of HDL-C may be related to a higher risk of ICH.

Table 1 outlines the details of major studies investigating the relationship between lipid levels and ICH. Most research show that lower TC and LDL-C and higher HDL-C are associated with the increasing risk of ICH. Although the precise mechanisms are still unclear, it was considered that low blood level of TC might contribute to an abnormal fragility of erythrocytes [22] and endothelial cells [23], necrosis of smooth muscle cell in arterial media [19], and angionecrosis [23]. But the relationship between TG and ICH is controversial. The inconsistent results might be due to the different participants involved in the researches or other factors. The subjects who participated in the Malmö Preventive Project [17] were younger and the follow-up period was shorter than those who participated in the projects which had opposite results. In Zhou’s cross-sectional study [18] TG level was tested after the acute ICH occurred, which was different from the prospective studies in which TG level was tested before the ICH occurred. Therefore, a higher TG level may be the result of stress [24] instead of a risk factor for ICH. These data suggest that the association between TG and ICH may be inconsistent in different individuals.

**Table 1:** Summary of studies investigating the relationship between lipid levels and ICH

Study	Study design	Sample size	Study population	Mean Age (y); Male (%)	Follow-Up in Years	Baseline lipid levels(mmol/L)	ICH (*n*)	Key study findings
[15] Sturgeon et al. 2007	pooled prospective cohort study (including 2 studies: ARIC and CHS)	21,680	without a history of stroke, American	54.2 (ARIC),72.8 (CHS); 44.8 % (ARIC), 42.4 % (CHS)	3 (ARIC); 5 (CHS)	TG: 1.49 (ARIC), 1.58 (CHS); TC: 5.56 (ARIC), 5.46 (CHS); LDL-C: 3.56 (ARIC), 3.36 (CHS); HDL-C: 1.33 (ARIC), 1.40 (CHS)	135	TG RR 0.56 (95 %CI 0.37–0.84); LDL-C (top 1/4 vs. lower 3/4): RR 0.52 (95 %CI 0.31–0.88)
[11] Bonaventure et al. 2009	population-based prospective cohort study	8393	without a history of stroke, French	≥65; 36.8 %	5	TC: 5.56 (TG ≤0.94), 5.81 (TG 0.95–1.3), 6.08 (TG ≥1.34); LDL-C: 1.82 (TG ≤0.94), 1.62 (TG 0.95–1.33), 1.39(TG ≥1.34); HDL-C: 1.82 (TG ≤0.94), 1.62 (TG 0.95–1.33), 1.39 (TG ≥1.34)	36	TG ≤0.94 mmol/L: adjusted HR 2.35 (95 %CI 1.18–4.70)
[16] Wieberdink et al. 2011	prospective cohort study	9068	without a history of stroke, Dutch	≥55; 57.1 %	9.7	TG, median (IQR): 1.3 (1.0–1.8); TC, median (IQR): 5.8 (5.2–6.5); LDL-C, median (IQR): 3.7 (3.2–4.3); HDL-C, median (IQR): 1.3 (1.1–1.6)	85; 162 CMB in 789 healthy participants	TG: HR 0.20 (95 %CI 0.06–0.69); TG (CMB): HR 0.37 (95 %CI 0.14–0.96)
[17] Zia et al. 2006	population-based nested case–control study	33,346 (1029 stroke-free controls, matched for age, sex and screening-year)	without a history of myocardial infarction or stroke, Swedish	47; 67.3 %	14	/	147	TG (among the lobar ICH): OR 1.7 (95 %CI 0.9–3.2); TG (among the nonlobar ICH):OR 1.4 (95 %CI 0.9–2.3)
[18] Zhou et al. 2003	cross-sectional study	700	without a history of stroke, Chinese	≥57	/	/	500	In ICH patients, TG and LDL-C were significantly increased (*P* < 0.0001), HDL-C was significantly decreased (*P* < 0.0001)
[13] O’Donnell et al. 2010	case–control study	4317	be admitted to hospital with first acute stroke whose causes were vascular, 22 countries	66.1; 63.0 %	3	/	663	TC OR 0.62 (99 %CI 0.42–0.92); HDL-C OR 1.91 (99 %CI 1.29–2.83); non-HDL-C: 0.50 (99 %CI 0.34–0.72)
[19] Wang et al. 2013	meta-analysis (19 prospective cohort studies, 4 nested case–control studies)	1,430,141	/	/	/	/	7960	high versus low analysis: TC RR 0.69 (95 %CI 0.59–0.81), LDL-C RR 0.62 (95 %CI 0.41–0.92), HDL-C RR 0.98 (95 %CI 0.80–1.19); dose–response analysis^a^: TC RR 0.85 (95 %CI 0.80–0.91), LDL-C RR 0.90 (95 %CI 0.77–1.05), HDL-C RR 1.11 (95 %CI 0.99–1.25)
[20] Suzuki et al. 2010	prospective cohort study	156,892	without a history of stroke, Japanese	53.3; 48.7 %	3	/	361	low TC (<160 mg/dl) was critical risks of ICH
[2] Romero et al. 2014	prospective cohort study	1965	without a history of stroke, American	66.5; 66.0 %	12	TC, mean (SD): 4.95 (0.96); LDL-C, mean (SD): 2.84 (0.83)	173	TC <10th percentile: 1.91 (95 %CI 1.20–3.03); LDL-C <10th percentile: 1.28 (95 %CI 0.75–2.19)
[12] Zhang et al. 2012	prospective cohort study	58,235	without a history of coronary heart disease or stroke, Finnish	25–74; 47.6 %	20.1	/	497	TC in women: <5 mmol/L HR 1.00, 5–5.9 mmol/L HR 0.58 (95 %CI 0.38–0.90), 6–6.9 mmol/L HR 0.40–0.94 (95 %CI 0.40–0.94), ≥7 mmol/L HR 0.50 (95 %CI 0.32–0.78); the relationship of TC and ICH in men was not significant
[21] Mustanoja et al. 2013	observational registry	964 (187 patients used statin before ICH)	Be admitted to hospital with ICH, Finnish	66; 57.1 %	/	TG, median (IQR): 1.0 (0.7–1.3); TC, median (IQR): 4.4 (3.8–5.1); LDL-C, median (IQR): 2.4 (1.8–3.0); HDL-C, median (IQR):1.4 (1.1.–1.8)	964	After adjusting for known ICH prognostic factors, lower LDL-C was independently associated with in-hospital mortality (OR 0.54, 95 %CI 0.31–0.93)

*ARIC* the atherosclerosis risk in communities study, *CHS* the cardiovascular health study, *IQR* interquartile range, *OR* odds ratio, *CI* confidence interval, *SD* standard deviation, *HR* hazard ratio, *RR* relative risk

^a^ for 1 mmol/L increment

## Statin therapy and ICH

Statins, also known as 3-hydroxy-3-methylglutaryl coenzyme A (HMG-CoA) reductase inhibitors, have been widely used clinically. A large number of studies has shown that statin therapy is an effective method for the treatment and prevention of atherosclerotic cardiovascular diseases. However, there is a concern whether lowering lipid levels by statin therapy may increase the risk of ICH. Table 2 outlines the details of major studies investigating the relationship between statin use and ICH. As shown in the Table 2, these results are not consistent.

**Table 2:** Summary of clinical studies investigating the relationship between statin use and ICH

Study	Study design	Sample size, *n* (statin group, *n*)	Population or settings	Statin	Dose	Mean age, y; male (%)	Follow-Up in years	ICH patients, *n* (statin group, *n*)	Outcome
[25] Dowlatshahi et al. 2012	case–control study	2466 (537 took statin before the occurrence of ICH, statins were discontinued on admission in 158 of 537)	be admitted to hospital with primary ICH, Canadian	No details	no details	71; 53.6 %	/	/	Compared with nonusers, statin users were less likely to have severe strokes (54.7 % vs. 63.3 %) but had similar rates of poor outcome (70 % vs. 67 %) and 30-day mortality (36 % vs. 37 %). The patients who discontinued statins on admission were more likely to have severe stroke (65 % vs. 27 %, *P* < 0.01), poor outcome (90 % vs. 62 %, *P* < 0.01)
[9] Flint et al. 2014	retrospective cohort study	3481 (1194)	be admitted to hospital with ICH, American	Lov, Sim, Ato	10 mg/d, in atorvastatin-equivalent dose	73.5; 50.1 %	No details	/	Improved 30-day survival: OR 4.25 (95 %CI 3.46–5.23)
[26] Pan et al. 2014	case–control study	3218 (220)	be admitted to hospital with ischemic stroke, ICH or TIA, Chinese	No details	a/	62.1; 61.2 %	1	/	Improved 3 months and 1 year survival: 3 months-survival: OR 2.24 (95 %CI 1.49–3.36); 1 year survival: OR 2.04 (95 %CI 1.37–3.06)
[7] Chen et al. 2014	population-based prospective cohort study	8333 (749)	be admitted to hospital with new-onset ICH, Taiwanese	Sim, Pra, Flu	20 mg/d, in atorvastatin-equivalent dose	59; 60.4 %	2	746 (69)	Did not increase the risk of recurrent ICH: adjusted HR 1.044 (95 %CI 0.812–1.341)
[33] Collins et al. 2004	randomized controlled trails	20,536 (10,269)	with a history of cardiovascular disease, other occlusive arterial disease, diabetes, or hypertension, British	Sim	40 mg/d	40–80; 75 %	4.8 (mean duration)	1029 (444)	No effect on ICH: OR 0.95 (95 %CI 0.65–1.40)
[34] Hackam et al. 2012	retrospective cohort study	17,872 (8936)	with a history of acute ischemic stroke, Canadian	No details	/	77.9; 46.3 %	4.2	213	No effect on ICH: HR 0.87 (95 %CI 0.65–1.17)
[35] Hackam et al. 2011	meta-analysis (23 randomized controlled trails, 12 cohort studies, 6 case–control studies, 1 case-crossover study)	248,391	Patients with atherosclerotic cardiovascular disease or risk factors for atherosclerosis, multicenter	No details	/	/,/	3.9 (IQR, 2.8–5.0)	14,784	No effect on ICH: random trials: OR 1.10 (95 % CI 0.86–1.14); cohort studies: OR 0.94 (95 % CI 0.81–1.10); case–control studies: OR 0.60 (95 % CI 0.41–0.88)
[36] McKinney et al. 2012	meta-analysis (31 randomized controlled trails)	182,803 (91,588 in the active group and 91,215 in the control group)	Patients with a history of diabetes mellitus, hypertension, cardiovascular disease, stroke or smoking, multicenter	/	/	62.6; 67.0 %	3.9 (median length)	676 (358 patients in the active group vs. 318 in the control group)	No effect on ICH: OR 1.08 (95 % CI 0.88–1.32)
[21] Mustanoja et al. 2013	observational registry	964 (187 patients used statin before ICH)	ICH patients, Finnish	No details	/	66; 57 %	No details	/	Premorbid statin use did not affect the outcome of ICH[in-hospital mortality: OR 1.11 (95 % CI 0.39–3.14); 3-month mortality: OR 1.57 (95 % CI 0.74–3.32); 12-month mortality: OR 0.97 (95 % CI 0.48–1.96)]
[14] Lei et al. 2014	meta-analysis (12 interventional or observational clinical studies)	6961(1652 patients used statin before ICH and 5309 nonusers	ICH patients, multicenter	Pra, Sim, Ato	10–40 mg/day	/,/	No details	2423 (569)^a^	No effect on in-hospital, 30-day and 90-day mortality: OR 0.85 (95 % CI 0.70–1.03)
[37] Amarenco et al. 2006	prospective random study	4731 (2365)	with a history of an ischemic or hemorrhagic stroke or a TIA, multicenter	Ato	80 mg/d	62.7; 59.6 %	4.9 (4.0–6.6)	88 (55)	5-year absolute reduction in the risk of fatal or nonfatal stroke: adjusted HR 0.84 (95%CI 0.71–0.99, *P* = 0.03)
[10] Goldstein et al. 2007^b^	the post hoc analysis of prospective random study	4731 (2365)	with a history of an ischemic or hemorrhagic stroke or a TIA, multicenter	Ato	80 mg/d	62.7; 59.6 %	4.9 (4.0–6.6)	88 (55)	Increased the risk of ICH: 2.3 % vs. 1.4 %, HR 1.68 (95 %CI 1.09–2.59)
[1] Scheitz et al. 2014	prospective cohort study	1446 (317 used statins before intravenous thrombolysis)	acute ischemic stroke patients receiving intravenous thrombolysis, American	Sim, Ato, Pra, Flu, Ros	20, 40, 80 mg/d, in simvastatin-equivalent dose	66.5; 66.0 %	/	53	Enhanced the risk of sICH: adjusted OR 2.4 (95 %CI 1.1–5.3) and 5.3 (95 %CI 2.3–12.3)^c^

*TIA* transient ischemic attack, *SPARCL* stroke prevention by aggressive reduction in cholesterol levels, *HR* hazard ratio, *OR* odds ratio, *RR* risk ratio, *Lov* lovastatin, *Sim* simvastatin, *Ato* atorvastatin, *Pra* pravastatin, *Flu* fluvastatin, *Ros* rosuvastatin

^a^ total events

^b^ a post hoc analysis of SPARCL study (Amarenco et al. [37])

^c^ for sICH for patients with medium or high-dose statins compared with non–statin users

### Statins may improve the outcome of ICH

After analyzing the clinical data of 2466 patients with ICH, Dowlatshahi et al. [25] found that the patients who took statin before the occurrence of ICH had lower risk of severe ICH compared with the nonusers (54.7 % vs. 63.3 %). In addition, among 537 statins users, 158 patients who discontinued its use on admission were more likely to have poor outcomes and higher 30-day mortality (71 % vs. 21 %, *P* < 0.01) compared with those who took statin continuously [25]. A retrospective cohort study showed that ICH patients who started statin therapy during hospitalization had a lower 30-day mortality rate than statin nonusers (18.4 % vs. 38.7 %, *P* < 0.001) and were more likely to be discharged to their homes or an acute rehabilitation facility (51.1 % vs. 22.3 %, *P* < 0.001) [9]. Pan et al. [26], using a multivariable logistic regression model adjusted for confounding factors, demonstrated that statin use during hospitalization for ICH patients was associated with more than 50 % lower mortality rate at 3 months (adjusted OR 0.44, 95 % CI 0.22–0.87, *P* < 0.001) and 1 year (adjusted OR 0.49, 95 % CI 0.27–0.86, *P* = 0.02) compared with those without statin therapy during admission.

It is believed statin may have the pleiotropic effects besides reducing serum lipid level. Using Magnetic Resonance Image (MRI) and immunohistochemistry, Yang et al. [27] found that simvastatin may protect the integrity of the blood–brain barrier (BBB) to prevent the dysfunction of it, resulting in the reduction of neurotoxic substances and white blood cells into the brain parenchyma [28, 29]. In addition, quantitative analysis showed simvastatin could improve the cerebral blood flow (CBF) and promote the functional recovery of cerebrum [27]. In experiments with rats, it was demonstrated that statins could reduce the activation of glial cells [30] and the release of the cytokines such as IL-1B and TNF-α by microglia and brain-derived neurotrophic factors [4], suppress phagocytosis of microglia [4], inhibit the oxidative stress and development of inflammatory reaction [30], decrease the cell necrosis around the hematoma [30, 31], and promote the recovery of the sensorimotor function [31]. Yang et al. [32] found that statins could also promote the hyperplasia of the ependymal lower cells in Wistar rats and the formation of synapses and increase vascular density in the hematoma boundary zone, resulting in a better functional recovery of the nervous system.

### Statins may be independent of ICH

A randomized and double-blinded trial [33], in which 20,536 adults with cerebrovascular disease, coronary heart disease, other occlusive arterial disease, diabetes or hypertension were randomly allocated 40 mg simvastatin daily or matching placebo, indicated that statin therapy was not associated with hemorrhagic stroke (0.5 % vs. 0.5 %, OR 0.95, 95 % CI 0.65–1.40, *P* = 0.8) during a mean 4.8 year follow-up period. A retrospective cohort study [34] involving 17,872 subjects aged 66 years and older who initiated statin therapy after acute ischemic stroke, demonstrated that there was no association between statin and ICH by the analysis comparing statin users with non-users (OR 0.87, 95 % CI 0.65–1.17). Hackam et al. [35] conducted a meta-analysis of 23 random trials and 19 observational studies (12 cohort studies, six case–control studies and one case-crossover study) which comprised 248,391 patients with atherosclerotic cardiovascular disease or risk factors for atherosclerosis, of which 14,784 were ICH patients. And the results showed there was no significant association between statin use and ICH in the random trials (OR 1.10, 95 % CI 0.86–1.14), cohort studies (OR 0.94, 95 % CI 0.81–1.10) and case–control studies (OR 0.60, 95 % CI 0.41–0.88). McKinney et al. [36] made a meta-analysis of 31 randomized controlled trials about statin therapy and a total of 182,803 subjects were included (91,588 in the active group and 91,215 in the control group). Three hundred and fifty-eight patients in the active group suffered ICH versus three hundred and eighteen in the control group, indicated that there is no association between statin and ICH (OR 1.08, 95 % CI 0.88–1.32, *P* = 0.47). Meta regression analysis demonstrated there was no relationship between the risk of ICH and (i) the degree of the LDL-C reduction (slope, 0.0043; SE, 0.0054; 95 % CI, −0.4831 to 0.0149; *P* = 0.43); (ii) the achieved level of LDL-C in the active treatment group (slope, −0.0049; SE, 0.0043; 95 % CI, −0.0133 to 0.0035; *P* = 0.26). In addition, the Helsinki ICH Study composed of 964 ICH patients [21] showed admission (23 % vs. 24 %, *P* = 0.785), 3 months (34 % vs. 32 %, *P* = 0.449) and 12 months (38 % vs. 36 %, *P* = 0.602) mortality did not differ between the patients who used statin before hospitalization and those who did not. It suggested that statin therapy before admission had no effect on the prognosis of ICH patients. Another meta-analysis of 12 studies which included 1652 patients who used statins before ICH and 5309 nonusers [14] demonstrated statin did not have a significant effect on the improvement of the admission, 30-day and 90-day mortality (OR 0.85, 95 % CI 0.70–1.03). The proportion of the patients receiving a Rankin score of 3–6 (OR 0.82, 95 % CI 0.60–1.11) or 4–6 (OR 0.79, 95 % CI 0.43–1.45) was not changed when premorbid statin was used. In other words, premorbid statin therapy did not improve the functional outcome significantly [14]. Furthermore, the volume of hematoma was not affected significantly with premorbid statin therapy [14]. A population-based prospective cohort study also revealed statin therapy initiated during admission or within 3 months after discharge did not increase the risk of recurrent ICH [7].

### Statins may promote ICH

The Stroke Prevention by Aggressive Reduction in Cholesterol Levels (SPARCL) study [37] is the only secondary prevention study by now to evaluate the effect of statin therapy in non-cardiac ischemic stroke or transient ischemic attack (TIA) associated with hypercholesterolemia. The study included 4731 patients, who had a history of stroke or TIA within 1–6 months and a serum LDL-C level of at least 100–190 mg/dl but did not have any known coronary heart disease. These patients were randomly allocated to the active group (80 mg atorvastatin daily) or control group (matched placebo). The results showed that decreased cholesterol level was associated with less risk of recurrent stroke. But the post hoc analysis [10] found that there were more hemorrhagic stroke patients in the treatment group than those in the placebo group (2.3 % vs. 1.4 %, HR 1.68, 95 % CI 1.09–2.59). Cox multivariable regression analysis showed that a hemorrhagic stroke as the entry event (HR 5.65, 95 % CI 2.82–11.30, *p* < 0.001), male (HR 1.79, 95 % CI 1.13–2.84, *P* = 0.01), age (10 y increments, HR 1.42, 95 % CI 1.16–1.74, *P* = 0.001) and having Stage 2 (JNC-7) hypertension (HR 6.19, 95 % CI 1.47–26.11, *P* = 0.01) were independently associated with the risk of hemorrhagic stroke. Scheitz, JF et al. [1] analyzed data from two European intravenous thrombolysis registries and found that 317 (22 %) used statins before intravenous thrombolysis among 1446 acute ischemic stroke patients and the frequency of symptomatic ICH was 2 %, 6 %, and 13 % in patients with low-, medium-, and high-dose statin (simvastatin 20, 40, 80 mg/d or equivalent) treatment, respectively (*P* < 0.01), which indicated that there was a correlation between the increasing dose of statin therapy and risk of ICH among patients who had been treated with simvastatin or atorvastatin.

These inconsistent results may be caused by different population and dosage of stains when the relationship between stain therapy and ICH was studied. As shown in Table 2, a majority of randomized controlled trials [33] and the meta-analyses [35, 36] which included the patients with a history of atherosclerotic cardiovascular disease or the subjects with risk factors for atherosclerosis (such as diabetes, hypertension or smoking) showed that statin use did not affect the occurrence or outcome of ICH. But certain patients with history of ICH or high risk of ICH appear to be at increased risk of brain hemorrhages when exposed to high-dose statin [1, 37]. Mustanoja et al. [21] reasoned that statin therapy could increase the risk of ICH because low serum cholesterol level may lead to the reduction of vessel resistance to tension, resulting in higher risk of rupture and hemorrhage.

## Conclusion

Although epidemiological investigation found that the population with lower serum TC and LDL-C levels had a higher risk of ICH, the reduction of blood lipid levels caused by statin therapy might not increase the risk of ICH. Based on the current data, we think statin use in the prevention and treatment of atherosclerotic cardiovascular disease does not increase the risk of ICH in most conditions. The pleiotropic effects of statins such as inhibition of inflammation and protection of BBB may improve the prognosis of acute hemorrhagic stroke patients. But for elderly patients who have history of ICH and poorly-controlled hypertension, statins especially used in high dose, may increase the risk of ICH. Therefore, patients with low TC and LDL-C and high risk of ICH should be cautious in the usage of statins.

## Abbreviations

ICH: intracerebral hemorrhage
sICH: symptomatic intracerebral hemorrhage
CMB: cerebral microbleed
TNF-α: tumor necrosis factor-α
IL-1β: interleukin-1β
BBB: blood–brain barrier
TC: total cholesterol
TG: triglyceride
LDL-C: low density lipoprotein cholesterol
HDL-C: high density lipoprotein cholesterol
ZO-1: zona occuludens-1
HAEC: human arterial endothelial cell
CBF: cerebral blood flow
